# [Expression of Concern] Valosin-containing protein promotes metastasis of osteosarcoma through autophagy induction and anoikis inhibition via the ERK/NF-κβ/beclin-1 signaling pathway

**DOI:** 10.3892/ol.2026.15806

**Published:** 2026-08-07

**Authors:** Xin Hua Long, Yun Fei Zhou, Min Lan, Shan Hu Huang, Zhi Li Liu, Yong Shu

Oncol Lett 18: 3823–3829, 2019; DOI: 10.3892/ol.2019.10716

Following the publication of the above paper, it was drawn to the Editor's attention by a concerned reader that, regarding the cell invasion assay data shown in Fig. 3B on p. 3826, the ‘Autophinib’ and ‘Spermidine’ data panels contained an overlapping section, such that data which were intended to show the results of differently performed experiments had apparently been derived from the same original source. Furthermore, upon performing an independent analysis of the data in this paper in the Editorial Office, it came to light that data panels in Fig. 3A, showing the results of the cell migration assay experiments, also contained a number of overlapping sections.

The authors have been contacted by the Editorial Office to offer an explanation for these apparent anomalies in the presentation of the data in this paper, although up to this time, no response from them has been forthcoming. Owing to the fact that the Editorial Office has been made aware of potential issues surrounding the scientific integrity of this paper, we are issuing an Expression of Concern to notify readers of this potential problem while the Editorial Office continues to investigate this matter further.

